# Phosphatase Wip1 in Immunity: An Overview and Update

**DOI:** 10.3389/fimmu.2017.00008

**Published:** 2017-01-17

**Authors:** Xiao-Fei Shen, Yang Zhao, Jin-Peng Jiang, Wen-Xian Guan, Jun-Feng Du

**Affiliations:** ^1^Department of General Surgery, Affiliated Drum Tower Hospital of Nanjing University Medical School, Nanjing, China; ^2^Transplantation Biology Research Division, Institute of Zoology, Chinese Academy of Sciences, Beijing, China; ^3^Department of Rehabilitation Medicine, PLA Army General Hospital, Beijing, China; ^4^Department of General Surgery, PLA Army General Hospital, Beijing, China

**Keywords:** wild-type p53-induced phosphatase 1, immunity, signaling pathways, tumorigenesis, inflammation

## Abstract

Wild-type p53-induced phosphatase 1 (Wip1) is a newly identified serine/threonine phosphatase, which belongs to the PP2C family. Due to its involvement in stress-induced networks and overexpression in human tumors, primary studies have mainly focused on the role of Wip1 in tumorigenesis. It now has also been implicated in regulating several other physiological processes such as organism aging and neurogenesis. Recent evidence highlights a new role of Wip1 in controlling immune response through regulating immune cell development and function, as well as through the interplay with inflammatory signaling pathways such NF-κB and p38 mitogen-activated protein kinase. In this short review, we will give an overview of Wip1 in immunity to better understand this important phosphatase.

## Introduction

Wild-type p53-induced phosphatase 1 (Wip1), which is a member of the PP2C (the type 2C family of protein phosphatases) family of phosphatases, is emerging as a direct p53 target in response to stresses (1). Based on the critical role of p53 in regulating tumor development, previous studies mainly focused on the role of Wip1 in controlling tumor transformation (2) and found that Wip1 can regulate tumorigenesis through attenuating several stress-induced kinases activities (3). With newly emerging functions of Wip1 identified, more and more recent studies also suggested the critical role of Wip1 in a wide range of conditions such as aging (4) and adult neurogenesis (2). Since Wip1-deficient mice presented abnormal lymphoid tissue structure and increased susceptibility to pathogens (5), the role of Wip1 in regulating immunity has also been investigated. In this review, we will give a brief introduction of Wip1 in regulating several signaling pathway networks in the context of immunity to deepen our knowledge on this important phosphatase.

## An Overview of Phosphatase Wip1

Wip1 was discovered in 1997 by Fiscella and is a direct p53 target, which is rapidly induced by ionizing radiation in p53-positive cells (1). Primary studies focused on the role of Wip1 in DNA damage repair and suggested that Wip1 serves as a negative feedback regulator for DNA damage checkpoints (6–8). Several key molecules within the DNA-damage response network have been shown to be direct targets of Wip1 including p38 mitogen-activated protein kinase (MAPK) (7), ataxia-telangiectasia mutated (ATM) (8, 9), and cell cycle checkpoint kinase 2 (10). It is also suggested that Wip1 is required for controlling H2A histone family in several cell lines after DNA damage *in vitro* (11–13). Since the function of DNA damage checkpoints is to preserve the genomic fidelity of proliferating cells and to prevent cellular transformation (6), the possible role of Wip1 in tumorigenesis is further studied. Wip1 deficiency can lead to the activation of p53, which plays a critical role in tumor suppression and the enhanced ATM/p53-mediated apoptosis (14), thereby resulting in significantly attenuated tumorigenesis in two tumor models including c-myc-induced lymphoma (14, 15) and adenomatosis polyposis coli (Min) intestinal tumorigenesis (3). The deletion of Wip1 can also protect mice from mammary tumorigenesis in MMTV-Erbb2 and MMTV-HRAS1 mice in a p38 MAPK-dependent manner (16, 17). Moreover, other types of oncogenes such as products of Cdkn2a gene, p16^lnk4a^, and p19^arf^ are also elevated in the absence of Wip1 and promote tumor suppression in mammary gland tumors. Consistent with these results, overexpression of Wip1 was reported in many human cancers (2). All these results further support the role of Wip1 in regulating tumorigenesis. Interestingly, Wip1 overexpression in mice does not lead to spontaneous tumor appearance (18), and Wip1 overexpression in the mammary gland epithelium is also not sufficient to induce cancer (2, 19). Therefore, Wip1 may actually not act as a cancer-initiating oncogene on its own but provides advantages for tumor development through its function on multiple target molecules. Despite substantial evidence over the last decade that defines Wip1 as an onco-protein, recent studies have also shed lights on the role of Wip1 in regulating other normal and/or pathophysiological process due to its wide range of substrates, such as autophagy (20), aging (21), adult neurogenesis (4), and liver regeneration (22).

## The Role of Wip1 in Immunity

### Wip1 in Immune Cell Development and Function

#### Neutrophils

Our group has identified the critical role of Wip1 in mastering the development and function of neutrophils. The expression of Wip1 is gradually upregulated during the differentiation of myeloid precursors into mature neutrophils with the highest expression in resting mature neutrophils, and it can negatively regulate the generation and homeostasis of neutrophils *in vivo* (23). Wip1-deficient mice displayed severe neutrophilia caused by the accelerated development of neutrophils in the bone marrow and higher CXCR2 (CXC chemokine receptor, CXCR) expression both on immature and mature neutrophils in the bone marrow. Higher expression of CXCR2 and lower expression of CXCR4 discovered in Wip1-deficient mice can drive the release of neutrophils from the bone marrow into the blood (23). Further mechanism studies showed that Wip1 negatively modulates the differentiation and maturation of neutrophils in a p38 MAPK-signal transducers and activators of transcription (STAT) 1-dependent manner (23). Moreover, the bactericidal activities and migration capacity of neutrophils are also tightly controlled by Wip1. The expression of Wip1 in human and mouse neutrophils was downregulated quickly after challenged by bacterial infection and pro-inflammatory cytokines (24). The downregulated expression of Wip1 was negatively related to the inflammatory cytokine production and bactericidal activities of neutrophils in a p38 MAPK-STAT1- and nuclear transcription factor (NF)-κB-dependent manner (24). Due to the importance of Wip1 in regulating neutrophil development and function, we also suggested that targets on Wip1 might be a new therapeutic strategy for the treatment of inflammatory bowel diseases (25) and intestinal ischemia reperfusion injury (26).

#### T Cells and Thymus

It was reported that young Wip1-deficient mice had smaller thymus, especially the thymic medulla. Analysis of thymocyte subset numbers showed that the number of TCRαβ [T cell receptor (TCR)]-positive thymocytes in Wip1-deficient mice was significantly decreased, while the number of TCRγδ-positive thymocytes remained normal (27). Detailed studies showed that there was no significant difference in the percentage of single positive (SP) thymocytes (CD4+ or CD8+) and double positive (DP) thymocytes (CD4+ CD8+) between wild-type and Wip1 knock out (KO) mice, but the number of these cells decreased. On the contrary, the number of thymocytes in the double negative (DN) stage (CD4− CD8−) is normal, while the ratio of DN thymocytes increased (27). These results indicated that thymocytes in Wip1-deficient mice displayed a defect in the process from DN stage to SP stage. Mechanism studies showed that the mRNA expression of Wip1 gradually increased during the process from DN3 (CD44− CD25+) stage to DN4 (CD44+ CD25−) stage, which resulted in the loss of inhibitory function of Wip1 on p53 protein activity. The upregulated activity of p53 promoted cell cycle arrest with more cells in the S/G2/M phase, leading to a decrease in the number of cells in the DN4 stage, DP stage, and SP stage (27). The signaling pathway p38 MAPK was not involved in this process as p38 inhibitor could not rescue the thymic phenotype (27). Therefore, these results suggested that Wip1 is required for normal TCRαβ-positive T cell development through downregulating p53 activation in the thymus.

As previously described, Wip1-deficient mice had smaller thymus, especially the thymic medulla. Despite the impaired development of T cells in the thymus of Wip1-deficient mice, the role of Wip1 in regulating thymic epithelial cells (TEC) has also been studied. Based on the localization and distinctive cell surface markers, TEC are divided into two subsets, cortical TEC (cTEC) and medullary TEC (mTEC) (28), both of which arises from a common biopotent progenitor cell (29). Wip1 deficiency in thymic epithelium selectively leads to the decreased mTEC/cTEC ratio, medullary thymic zone, and the percentage and total cell number of mTEC (30), all of which indicates a defect in mTEC differentiation. Further studies showed that Wip1 controls mTEC developmental kinetics during mTEC maturation but not the fetal stage through the p38 MAPK/CD40 pathway as Wip1 deficiency does not affect the pool of thymic epithelia precursor cell, and the ratio of mature-to-immature mTEC is decreased in Wip1-deficient mice (30).

#### B Cells

The development of B cells is composed of several stages, pro-B cell, pre-B cell, and immature and mature B cell (31, 32). Wip1-deficient mice exhibit a remarkable reduction of B-cell numbers in the bone marrow, peripheral blood, and spleen (33). Through the establishment of mixed chimerism, studies showed that the decreased number of B cells found in Wip1-deficient mice was mainly caused by the impaired development of B cells during the pre-B-cell stage as Wip1-deficient bone marrow cells (BMCs) differentiated into B cells in a lower efficiency compared with the WT BMCs and Wip1 deficiency also led to more cell death in pre-B cells (33). Since DNA recombination events induce DNA damage response which leads to p53 activation and apoptosis during B-cell development (34, 35), the activation of p53 results in the upregulation of Wip1 which could dampen p53 protein level (33). Therefore, Wip1 serves as a negative feedback regulator to maintain the homeostatic level of p53 to support B-cell development, especially at the pre-B-cell stage. Moreover, Wip1 is also suggested to play a critical role in preventing an aging-related decline in B-cell development as Wip1 deficiency exacerbates the impairment of the pre-B-cell development both in physiological aging and experimentally forced replicative aging (33).

#### Macrophages

**Figure 1:**
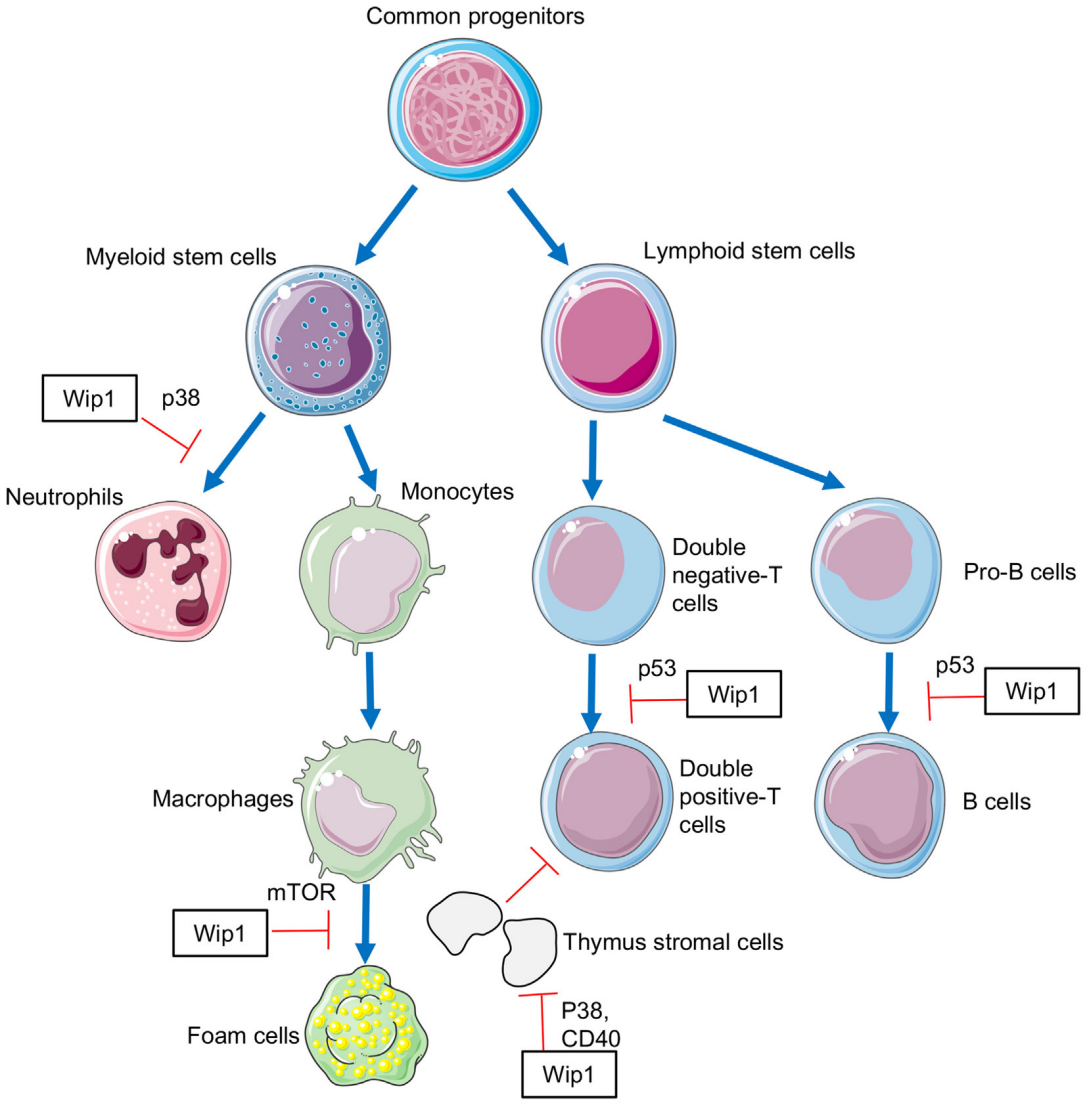
**The involvement of Wip1 in the development of immune cells**.

Study on the role of Wip1 in regulating the development of macrophages, as well as in the polarization of macrophages, is rare. However, in a recent study, Wip1 is suggested to play a critical role in regulating the conversion of macrophages into foam cells (20). In an apoE^−/−^ mouse model of atherosclerosis, Wip1 deficiency resulted in a reduction in atherosclerotic plaque formation (20), which is mainly dependent on formation of foam cells (36). Consistent with these results, *in vitro* studies demonstrated that Wip1 deficiency led to a markedly reduced accumulation of foam cells from macrophages, as well as a significant reduction in the accumulation of cholesterylesters in macrophages (20). Further mechanism studies showed that deletion of Wip1 led to ATM-dependent activation of initiation and progression of autophagy in macrophages, which suppressed the conversion of macrophages into foam cell, thus preventing the development of atherosclerosis (20) (Figure 1).

## Wip1 in Inflammation

**Figure 2:**
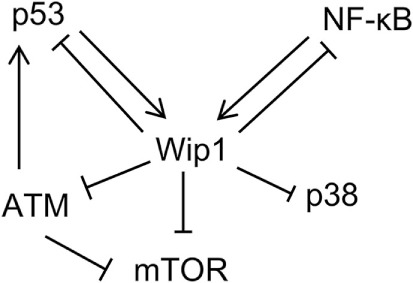
**Schematic depicting signaling pathway networks of Wip1 and other relative proteins**.

Primary evidence for the involvement of Wip1 in inflammation comes from the study, which showed that Wip1-deficient mice presented abnormal lymphoid structure, with increased susceptibility to ulcerated skin lesions and pathogens (5). We further demonstrated that Wip1 can modulate neutrophil function to regulate intestinal ischemia/reperfusion injury (26, 37) and inflammatory bowel diseases in a p38 MAPK-dependent manner (25). Moreover, Wip1 can also participate in neuro-inflammation through controlling tumor necrosis factor (TNF)-α synthesis (38) or NF-κB function (39). Further studies identified the interplay between Wip1 and NF-κB which plays an important role in cellular inflammatory response. NF-κB can activate *WIP1* gene expression. The *WIP1* gene has a conserved κB-binding site and the upregulation and inhibition of NF-κB function leads to increased and decreased Wipl expression, respectively. It was confirmed that the p65 subunit of NF-κB could directly bind to the upstream promoter of *WIP1* gene to regulate its biological activity. On the other hand, Wipl can negatively regulate the expression of NF-κB. The inhibition of Wip1 can lead to the enhanced activation of NF-κB. The mRNA expression of NF-κB target molecules such as TNF-α in Wip1-deficient HeLa cells was increased, and the levels of NF-κB-dependent pro-inflammatory cytokines were also increased in Wip1-deficient splenic cells. Moreover, splenocytes in Wip1-deficient mice presented a pro-inflammatory phenotype with increased ratio of cells expression CD80, MHC II, and CD40. On the contrary, HeLa cells with overexpression of Wip1 expressed much more decreased level of NF-κB-dependent cytokines. Detailed mechanism studies further showed that Wipl can inhibit the recruitment of NF-κB to co-transcription factor p300 by dephosphorylating P65 (Ser536) of NF-κB, leading to the inability of NF-κB to effectively activate the downstream pathway. In addition, Wipl can also inhibit the function of NF-κB by negative regulation of p38, resulting in reduced expression of NF-κB-dependent inflammatory factors such as IL-1, 6, and 8. Therefore, mechanism for Wip1 in regulating inflammation can be described as follows: the activation of NF-κB by inflammatory signals can increase the expression of Wip1, which acts as a negative feedback regulator to inhibit NF-κB signaling and maintain the cell homeostasis through turning off the inflammatory process (Figure 2).

## Concluding Remarks

Wip1 has long been recognized as an oncotarget, and therapeutic strategies to modulate its activity such as the use of Wip1 inhibitor have also been suggested to be a promising treatment for patients with tumors. Despite the intrinsic interplay between Wip1 and other signaling pathways in tumor cells, substantial experimental evidence has identified the critical role of Wip1 in regulating immunity through mastering immune cell development and function through numerous signaling pathways including p38 MAPK, STAT1, NF-κB, and ATM/p53. Although there is no direct evidence for Wip1 in controlling immune system to promote tumor development, it is possible that Wip1 can modulate tumor transformation through regulating immune system because of the tight relationship between inflammation and tumorigenesis.

Despite in regulating in immune system and tumorigenesis, Wip1 also displays a broader function in aging, liver regeneration, and adult neurogenesis. All these facts raise the concern that although Wip1 can regulate numerous molecular networks, its function largely depends on cell type, the condition, and the age of organism. Moreover, due to its potent anti-inflammatory and anti-aging function, strategies to increase Wip1 expression to control inflammation and aging should also take into account possible pro-tumorigenic effect of Wip1 activation. Therefore, future studies on understanding the crosstalk between Wip1 and multiple signaling pathways in different cells, as well as in whole organism level (i.e., immune system and secretion), may help to better develop potential therapeutic strategies based on phosphatase Wip1.

## Author Contributions

J-FD and W-XG suggested this topic; YZ and J-PJ retrieved the relevant literature; J-FD and X-FS wrote the paper.

## Conflict of Interest Statement

The authors declare that the research was conducted in the absence of any commercial or financial relationships that could be construed as a potential conflict of interest.

## References

[1] Fiscella M Zhang H Fan S Sakaguchi K Shen S Mercer WE Wip1, a novel human protein phosphatase that is induced in response to ionizing radiation in a p53-dependent manner. Proc Natl Acad Sci U S A (1997) 94(12):6048–53. 10.1073/pnas.94.12.6048 9177166 PMC20998

[2] Zhu YH Bulavin DV. Wip1-dependent signaling pathways in health and diseases. Prog Mol Biol Transl Sci (2012) 106:307–25. 10.1016/B978-0-12-396456-4.00001-8 22340722

[3] Demidov ON Timofeev O Lwin HN Kek C Appella E Bulavin DV. Wip1 phosphatase regulates p53-dependent apoptosis of stem cells and tumorigenesis in the mouse intestine. Cell Stem Cell (2007) 1(2):180–90. 10.1016/j.stem.2007.05.020 18371349

[4] Zhu Y Demidov ON Goh AM Virshup DM Lane DP Bulavin DV. Phosphatase WIP1 regulates adult neurogenesis and WNT signaling during aging. J Clin Invest (2014) 124(7):3263–73. 10.1172/JCI73015 24911145 PMC4071391

[5] Choi J Nannenga B Demidov ON Bulavin DV Cooney A Brayton C Mice deficient for the wild-type p53-induced phosphatase gene (Wip1) exhibit defects in reproductive organs, immune function, and cell cycle control. Mol Cell Biol (2002) 22(4):1094–105. 10.1128/MCB.22.4.1094-1105.2002 11809801 PMC134641

[6] Sancar A Lindsey-Boltz LA Unsal-Kacmaz K Linn S. Molecular mechanisms of mammalian DNA repair and the DNA damage checkpoints. Annu Rev Biochem (2004) 73:39–85. 10.1146/annurev.biochem.73.011303.073723 15189136

[7] Takekawa M Adachi M Nakahata A Nakayama I Itoh F Tsukuda H p53-inducible wip1 phosphatase mediates a negative feedback regulation of p38 MAPK-p53 signaling in response to UV radiation. EMBO J (2000) 19(23):6517–26. 10.1093/emboj/19.23.6517 11101524 PMC305857

[8] Batchelor E Mock CS Bhan I Loewer A Lahav G. Recurrent initiation: a mechanism for triggering p53 pulses in response to DNA damage. Mol Cell (2008) 30(3):277–89. 10.1016/j.molcel.2008.03.016 18471974 PMC2579769

[9] Shreeram S Demidov ON Hee WK Yamaguchi H Onishi N Kek C Wip1 phosphatase modulates ATM-dependent signaling pathways. Mol Cell (2006) 23(5):757–64. 10.1016/j.molcel.2006.07.010 16949371

[10] Fujimoto H Onishi N Kato N Takekawa M Xu XZ Kosugi A Regulation of the antioncogenic Chk2 kinase by the oncogenic Wip1 phosphatase. Cell Death Differ (2006) 13(7):1170–80. 10.1038/sj.cdd.4401801 16311512

[11] Cha H Lowe JM Li H Lee JS Belova GI Bulavin DV Wip1 directly dephosphorylates gamma-H2AX and attenuates the DNA damage response. Cancer Res (2010) 70(10):4112–22. 10.1158/0008-5472.CAN-09-4244 20460517 PMC2904079

[12] Moon SH Nguyen TA Darlington Y Lu X Donehower LA Dephosphorylation of gamma-H2AX by WIP1: an important homeostatic regulatory event in DNA repair and cell cycle control. Cell Cycle (2010) 9(11):2092–6. 10.4161/cc.9.11.11810 20495376 PMC3984036

[13] Macurek L Lindqvist A Voets O Kool J Vos HR Medema RH. Wip1 phosphatase is associated with chromatin and dephosphorylates gammaH2AX to promote checkpoint inhibition. Oncogene (2010) 29(15):2281–91. 10.1038/onc.2009.501 20101220

[14] Shreeram S Hee WK Demidov ON Kek C Yamaguchi H Fornace AJ Jr Regulation of ATM/p53-dependent suppression of myc-induced lymphomas by Wip1 phosphatase. J Exp Med (2006) 203(13):2793–9. 10.1084/jem.20061563 17158963 PMC2118180

[15] Lu X Nannenga B Donehower LA. PPM1D dephosphorylates Chk1 and p53 and abrogates cell cycle checkpoints. Genes Dev (2005) 19(10):1162–74. 10.1101/gad.1291305 15870257 PMC1132003

[16] Harrison M Li J Degenhardt Y Hoey T Powers S. Wip1-deficient mice are resistant to common cancer genes. Trends Mol Med (2004) 10(8):359–61. 10.1016/j.molmed.2004.06.010 15310454

[17] Bulavin DV Phillips C Nannenga B Timofeev O Donehower LA Anderson CW Inactivation of the Wip1 phosphatase inhibits mammary tumorigenesis through p38 MAPK-mediated activation of the p16(Ink4a)-p19(Arf) pathway. Nat Genet (2004) 36(4):343–50. 10.1038/ng1317 14991053

[18] Goloudina AR Kochetkova EY Pospelova TV Demidov ON. Wip1 phosphatase: between p53 and MAPK kinases pathways. Oncotarget (2016) 7(21):31563–71. 10.18632/oncotarget.7325 26883196 PMC5058778

[19] Demidov ON Kek C Shreeram S Timofeev O Fornace AJ Appella E The role of the MKK6/p38 MAPK pathway in Wip1-dependent regulation of ErbB2-driven mammary gland tumorigenesis. Oncogene (2007) 26(17):2502–6. 10.1038/sj.onc.1210032 17016428

[20] Le Guezennec X Brichkina A Huang YF Kostromina E Han W Bulavin DV. Wip1-dependent regulation of autophagy, obesity, and atherosclerosis. Cell Metab (2012) 16(1):68–80. 10.1016/j.cmet.2012.06.003 22768840

[21] Chen Z Yi W Morita Y Wang H Cong Y Liu JP Wip1 deficiency impairs haematopoietic stem cell function via p53 and mTORC1 pathways. Nat Commun (2015) 6:6808. 10.1038/ncomms7808 25879755

[22] Zhang L Liu L He Z Li G Liu J Song Z Inhibition of wild-type p53-induced phosphatase 1 promotes liver regeneration in mice by direct activation of mammalian target of rapamycin. Hepatology (2015) 61(6):2030–41. 10.1002/hep.27755 25704606

[23] Liu G Hu X Sun B Yang T Shi J Zhang L Phosphatase Wip1 negatively regulates neutrophil development through p38 MAPK-STAT1. Blood (2013) 121(3):519–29. 10.1182/blood-2012-05-432674 23212517

[24] Sun B Hu X Liu G Ma B Xu Y Yang T Phosphatase Wip1 negatively regulates neutrophil migration and inflammation. J Immunol (2014) 192(3):1184–95. 10.4049/jimmunol.1300656 24395919

[25] Hu X Wang P Du J Yang F Tian Y Shen X Phosphatase Wip1 masters IL-17-producing neutrophil-mediated colitis in mice. Inflamm Bowel Dis (2016) 22(6):1316–25. 10.1097/MIB.0000000000000751 26950306

[26] Shen X Du J Zhao Y Guan W. Phosphatase Wip1 as a new therapeutic target for intestinal ischemia-reperfusion injury. Expert Rev Clin Immunol (2014) 10(12):1591–5. 10.1586/1744666X.2014.975211 25382183

[27] Schito ML Demidov ON Saito S Ashwell JD Appella E. Wip1 phosphatase-deficient mice exhibit defective T cell maturation due to sustained p53 activation. J Immunol (2006) 176(8):4818–25. 10.4049/jimmunol.176.8.4818 16585576

[28] Manley NR Condie BG. Transcriptional regulation of thymus organogenesis and thymic epithelial cell differentiation. Prog Mol Biol Transl Sci (2010) 92:103–20. 10.1016/S1877-1173(10)92005-X 20800818

[29] Bleul CC Corbeaux T Reuter A Fisch P Monting JS Boehm T. Formation of a functional thymus initiated by a postnatal epithelial progenitor cell. Nature (2006) 441(7096):992–6. 10.1038/nature04850 16791198

[30] Sun L Li H Luo H Zhang L Hu X Yang T Phosphatase Wip1 is essential for the maturation and homeostasis of medullary thymic epithelial cells in mice. J Immunol (2013) 191(6):3210–20. 10.4049/jimmunol.1300363 23966632

[31] Hardy RR Hayakawa K B cell development pathways. Annu Rev Immunol (2001) 19:595–621. 10.1146/annurev.immunol.19.1.595 11244048

[32] Reth M Nielsen P. Signaling circuits in early B-cell development. Adv Immunol (2014) 122:129–75. 10.1016/B978-0-12-800267-4.00004-3 24507157

[33] Yi W Hu X Chen Z Liu L Tian Y Chen H Phosphatase Wip1 controls antigen-independent B-cell development in a p53-dependent manner. Blood (2015) 126(5):620–8. 10.1182/blood-2015-02-624114 26012568 PMC4520877

[34] Lu L Lejtenyi D Osmond DG. Regulation of cell survival during B lymphopoiesis: suppressed apoptosis of pro-B cells in P53-deficient mouse bone marrow. Eur J Immunol (1999) 29(8):2484–90. 10.1002/(SICI)1521-4141(199908)29:08<2484::AID-IMMU2484>3.0.CO;2-B 10458762

[35] Lu L Chaudhury P Osmond DG. Regulation of cell survival during B lymphopoiesis: apoptosis and Bcl-2/Bax content of precursor B cells in bone marrow of mice with altered expression of IL-7 and recombinase-activating gene-2. J Immunol (1999) 162(4):1931–40. 9973461

[36] Glass CK Witztum JL Atherosclerosis: the road ahead. Cell (2001) 104(4):503–16. 10.1016/S0092-8674(01)00238-0 11239408

[37] Du J Shen X Zhao Y Hu X Sun B Guan W Wip1-deficient neutrophils significantly promote intestinal ischemia/reperfusion injury in mice. Curr Mol Med (2015) 15(1):100–8. 10.2174/1566524015666150114122929 25601473

[38] Tan X Zhang J Jin W Li L Xu W Zheng H Wip1 phosphatase involved in lipopolysaccharide-induced neuroinflammation. J Mol Neurosci (2013) 51(3):959–66. 10.1007/s12031-013-0080-y 23959423

[39] Zhong H Cui L Xu F Chen L Jiang L Huang H Up-regulation of Wip1 involves in neuroinflammation of retinal astrocytes after optic nerve crush via NF-kappaB signaling pathway. Inflamm Res (2016) 65(9):709–15. 10.1007/s00011-016-0952-z 27207279

